# Associations of lower-limb muscle strength performance with static and dynamic balance control among older adults in Taiwan

**DOI:** 10.3389/fpubh.2024.1226239

**Published:** 2024-02-13

**Authors:** Ping-Chun Yeh, De-Kai Syu, Chien-Chang Ho, Tian-Shyug Lee

**Affiliations:** ^1^Graduate Institute of Business Administration, Fu Jen Catholic University, New Taipei City, Taiwan; ^2^Sports Medicine Center, Fu Jen Catholic Hospital, New Taipei City, Taiwan; ^3^Department of Physical Education, Fu Jen Catholic University, New Taipei City, Taiwan; ^4^Research and Development Center for Physical Education, Health and Information Technology, College of Education, Fu Jen Catholic University, New Taipei City, Taiwan; ^5^Artificial Intelligence Development Center, Fu Jen Catholic University, New Taipei City, Taiwan

**Keywords:** lower-limb muscle strength, balance, older adult, cross-sectional study, Taiwan

## Abstract

**Background:**

Aging is an inevitable process of life development. These physical changes can cause a decline in the functional adaptability and health status of older adult individuals.

**Aims:**

The purpose of this study was to investigate the association of lower-limb muscle strength performance with static and dynamic balance control among older adults in Taiwan.

**Methods:**

We conducted a cross-sectional study and reviewed data derived from the National Physical Fitness Survey in Taiwan 2015–2016. A total of 20,846 Taiwanese older adult individuals aged 65 years old or older were recruited as study participants. Demographic characteristics, anthropometric assessments, lifestyle habits, and health-related physical fitness measurements from this dataset were analyzed using the chi-square test, one-way analysis of variance, and linear regression analysis. Lower-limb muscle strength performance was classified into 4 groups based on quartile (Quartile 1 [Q1], Quartile 2 [Q2], Quartile 3 [Q3], and Quartile 4 [Q4]) values.

**Results:**

Increased lower-limb muscle strength levels were significantly associated with static balance in men (Q2: *β* = 2.539, *p* < 0.0001; Q3: *β* = 4.590, *p* < 0.0001; Q4: *β* = 7.700, *p* < 0.0001) and women (Q2: *β* = 2.022, *p* < 0.0001; Q3: *β* = 4.179, *p* < 0.0001; Q4: *β* = 6.920, *p* < 0.0001) after adjusting for potential confounders. In addition, we observed that increased lower-limb muscle strength levels were significantly associated with dynamic balance in men (Q2: *β* = −1.661, *p* < 0.0001; Q3: *β* = −2.434, *p* < 0.0001; Q4: *β* = −3.091, *p* < 0.0001) and women (Q2: *β* = −1.660, *p* < 0.0001; Q3: *β* = −2.548, *p* < 0.0001; Q4: *β* = −3.196, *p* < 0.0001) after adjusting for potential confounders.

**Conclusion:**

Lower-limb muscle strength was the most important factor, as it was an improved method for static and dynamic balance control in both genders.

## Introduction

In 2022, there were 4.1 million or 17.5% adults older than 65 years old living in Taiwan (1). Furthermore, it is estimated that the proportion of Taiwanese older adults could be higher than 20% by 2025, which refers to a “superaged society.” Aging is strongly associated with a decrease in muscle strength and muscle mass, resulting in physical dysfunction (e.g., gait speed agility and balance), falling and deterioration in quality of life (2, 3). Therefore, successful aging is an important issue for the older adult population in Taiwan to promote health and maintain active engagement with life (4, 5).

Falls are the leading cause of fractures and even deaths among older adult adults (6). The most common fall-induced fractures occur at the hip, spine, upper arm and forearm (7). Of these fractures, hip fractures have serious consequences, such as reduced mobility and independence and high mortality in the first three months (3, 8). Numerous studies indicated that older adult fallers are characterized by lower muscle strength of the lower extremities and slower walking speeds (6, 9–12). A similar study reported that the main determinants for recurrent falls included fall history, muscle strength, abnormal postural sway (e.g., balance and gait disturbances), and depression (8, 13–15).

It has been shown that the one-leg stance with eye open test is a readily applicable screening tool in clinical and geriatric environments, used to assess postural steadiness during static positions (16); failing to pass a 5-s threshold could indicate functional impairment, thereby heightening the risk of falls (17). Khanal et al. suggested that older adult individuals with a one-leg standing test below the 55-s threshold (the sum of both legs) have low muscle mass and a high risk of developing sarcopenia (18). A study indicated that independent older adult adults have higher walking speeds than dependent older adults, such as those living in nursing or rest homes (19). In addition, the 8-foot up-and-go test has been successfully associated with dynamic balance, agility and walking speed performance, as well as fall risk, in the older adult population (2). Thus, older adult adults with good static and dynamic balance and quick walking speeds might have better independence and a lower risk of sarcopenia and falling. This was supported by past research, which found that the risk of falls in the older adult was associated with low muscle strength and physical fitness, but not with low muscle mass (20).

Taiwan is not only a highly technologically developed country but also an aged society. To our knowledge, no study has yet reported the relationship between lower extremity muscle strength and static and dynamic balance among Taiwanese older populations. Therefore, in the present study, we aimed to investigate the association between lower-limb muscle strength performance and static and dynamic balance control among older adults in Taiwan.

## Materials and methods

### Study design and sample

This study used a cross-sectional study design with data from the National Physical Fitness Survey in Taiwan (NPFSIT) 2015–2016. The NPFSIT was conducted by the Sports Administration, Ministry of Education in Taiwan and assessed the health-related physical fitness status of a sample population of community-dwelling older Taiwanese adults (≥ 65 years old) selected according to a convenience sampling. Convenience sampling is a specific type of non-probabilistic sampling method that relies on data collection from populations who are conveniently available to participate in the study, and it is most frequently used in quantitative studies (21).

A total of 21,386 participants in the NPFSIT 2015–2016 were recruited from 35 testing stations in 20 cities or counties in Taiwan from October 2015 to April 2016; the detailed procedure of the present study has been published elsewhere (22–24). The NPFSIT combines face-to-face interviews (demographic and socioeconomic characteristics, health-related lifestyles, and disease history) and health-related physical fitness surveys with resting heart rate and blood pressure measurements for safe preliminary screening before conducting physical fitness tests. Table 1 presents the demographic background characteristics of the 20,846 older adults classified into four quartiles of lower limb muscle strength performance. The participants’ demographic characteristics included age, gender, body composition (height, body weight, body mass index [BMI], waist circumference [WC], hip circumference [HC], waist-to-hip ratio [WHR]), education level, income level, marital status, relationship status, and regular exercise.

**Table 1:** Characteristics of the study participants according to lower-limb muscle strength levels among older adults in Taiwan.

Variables	Lower-limb muscle strength levels				*F*/*x*^2^ (*p*)	Tukey’s *post hoc* test
	Quartile 1 (*n* = 4,394)	Quartile 2 (*n* = 5,142)	Quartile 3 (*n* = 5,702)	Quartile 4 (*n* = 5,608)		
Age (%)	76.54 ± 6.74	74.22 ± 6.33	72.71 ± 6.00	71.50 ± 5.49	627.74 (< 0.0001*)	Q1 > Q2 > Q3 > Q4
65–74 years old	40.10	55.06	64.91	74.14	1444.49 (< 0.0001*)	
75–84 years old	47.09	37.94	31.09	23.20		
≧85 years	12.81	7.00	4.00	2.66		
Gender (%)					(< 0.0001*)	
Male	39.96	35.80	27.85	39.21		
Female	60.04	64.20	72.15	60.79		
Height (cm)	156.03 ± 8.20	156.36 ± 7.85	155.57 ± 7.53	156.78 ± 7.92	23.77 (< 0.0001*)	Q3 < Q1, Q2 < Q4
Body weight (kg)	61.48 ± 10.79	61.44 ± 10.55	59.92 ± 9.96	59.76 ± 9.87	42.93 (< 0.0001*)	Q1, Q2 > Q3, Q4
BMI (kg/m^2^)	25.21 ± 3.75	25.09 ± 3.66	24.72 ± 3.52	24.27 ± 3.31	73.28 (< 0.0001*)	Q1, Q2 > Q3 > Q4
WC (cm)	89.03 ± 10.09	87.19 ± 9.70	84.98 ± 9.64	83.44 ± 9.17	324.63 (< 0.0001*)	Q1 > Q2 > Q3 > Q4
HC (cm)	97.12 ± 7.57	96.63 ± 7.52	95.74 ± 7.08	94.16 ± 6.48	121.99 (< 0.0001*)	Q1 > Q2 > Q3 > Q4
WHR	0.92 ± 0.07	0.90 ± 0.07	0.89 ± 0.07	0.88 ± 0.07	252.14 (< 0.0001*)	Q1 > Q2 > Q3 > Q4
Education level (%)					1220.16 (< 0.0001*)	
Elementary school or lower	77.49	69.55	61.25	45.62		
Junior or senior school	17.23	22.01	27.06	35.27		
College or higher	5.29	8.44	11.70	19.12		
Monthly income level (%)					295.31 (< 0.0001*)	
≦ 20,000 NTD	93.13	90.81	88.48	82.56		
20,001–40,000 NTD	5.07	5.76	6.90	9.72		
≧ 40,001 NTD	1.80	3.43	4.61	7.72		
Marital status (%)					99.41 (< 0.0001*)	
Never married	4.33	3.19	2.95	3.89		
Married	74.99	79.82	80.45	82.53		
Divorced/separation/widowed	20.69	16.98	16.60	13.58		
Relationship status (%)					46.36 (< 0.0001*)	
Living with someone	78.70	81.27	81.49	84.07		
Not living with someone	21.30	18.73	18.51	15.93		
Weekly regular exercise level (%)					40.57 (< 0.0001*)	
≧150 min/week	21.03	24.17	25.41	26.23		
< 150 min/week	78.97	75.83	74.59	73.77		
30-s chair stand (reps)	8.38 ± 2.53	12.39 ± 0.92	15.52 ± 1.05	21.49 ± 3.67	28189.40 (< 0.0001*)	Q1 < Q2 < Q3 < Q4

BMI, body mass index; HC, hip circumference; NTD, new Taiwan dollar; Quartile 1, Q1; Quartile 2, Q2; Quartile 3, Q3; Quartile 4, Q4; SD, standard deviation; WC, waist circumference; WHR, waist-to-hip ratio. Values are expressed as the means ± SDs. ^*^p < 0.05.

Detailed information about NPFSIT is available at https://isports.sa.gov.tw/index.aspx. Ethical procedures for the study were approved by the Institutional Review Board of Fu Jen Catholic University (FJU-IRB C110113), and written informed consent was obtained from each participant.

### Eligibility criteria for study participants

The target population for the NPFSIT was the 2,866,067 older adults whose residence were registered in Taiwan in the middle of 2015 (25). Subsequently, the representative sample size with stratification by gender and age needed, to achieve the study objectives and sufficient statistical power, was calculated with a sample size calculator (26). The sample size calculator arrived at 1,848 participants for each gender- and age-stratification, using a margin of error of ±3%, and a confidence level of 99%. The required total sample size was 18,480 participates in the NPFSIT 2015–2016. The exclusion criteria were (1) systolic blood pressure ≥ 140 mmHg or diastolic blood pressure ≥ 90 mmHg; and (2) currently/previously have heart disease, hypertension, chest pain, vertigo or musculoskeletal disorders. Finally, our analysis included a final total of 20,846 Taiwanese older adult participants.

### Lower extremity muscle strength performance

Lower extremity muscle strength performance was determined using the 30-s chair stand test (reps). For the 30-s chair stand test, each participant had to sit on a chair with the hands on the opposite shoulder crossed at the wrists, feet flat on the ground, and back straight. At the “Start” sign, individuals had to rise to a full standing position and sit back down again as fast as they could for 30 s. Participants performed the test once, and the total number of repetitions within 30 s was recorded for analysis. Jones et al. (27) reported that the correlation between the 30-s chair stand test and one-repetition maximum muscle strength is relatively high (*r* = 0.77). In addition, the high reliability of 30-s chair stand test is 0.89 and considered suitable for demonstrating a reliable and valid measurement of lower extremity muscle strength (27, 28).

The analysis of continuous variables for lower extremity muscle strength performance were conducted. Furthermore, lower extremity muscle strength levels were then categorized into quartiles of 30-s chair stand test performance within gender category strata, with Quartile 1 (Q1) having the worst and Quartile 4 (Q4) the best performance. The quartiles of 30-s chair stand test in males were < 12 reps (Q1), 12–14 reps (Quartile 2 [Q2]), 15–18 reps (Quartile 3 [Q3]), and > 18 reps (Q4). The quartiles of 30-s chair stand test in females were < 11 reps (Q1), 11–13 reps (Q2), 14–18 reps (Q3), and > 18 reps (Q4).

### Balance control

Two types of balance control were considered in this study. Static balance was determined using a one-leg stance with an open eye test (seconds). One-leg stance with eye open was measured on the preferred leg indicated by each participant. Individuals had to place their hands on their hips, lift the nonpreferred leg off the ground and maintain their balance while standing with the preferred leg. The participant was timed starting from when the nonpreferred leg was raised until the nonpreferred leg was set back down on the floor, the crossed hands were separated from their hips, or the preferred leg moved from the standing position. Performance was quantified in seconds, a time that took the participant to complete the test. In addition, the high reliability of one-leg stance with an open eye test is 0.90–0.91 and considered suitable for demonstrating a reliable and valid measurement of static balance (29).

Dynamic balance was determined using the 8-foot up-and-go test (seconds). The test began with the participant fully seated on a chair placed at the starting point. On the “Start” signal, the individual rose from the chair, walked around a cone that was placed 8 feet (2.44 m) from the chair, and returned to a seated position on the chair as fast as possible. Performance was quantified in seconds, i.e., a time that the participant took to complete the test. In addition, the high reliability of 8-foot up-and-go test is −0.81 and considered suitable for demonstrating a reliable and valid measurement of dynamic balance (30).

All participants performed the static and dynamic balance tests twice, and better performance was recorded for analysis.

### Covariate assessment

Considering the impact of other covariates on static and dynamic balance control performance, we controlled for potential confounders to perform covariate-adjusted analyses. The selection of potential confounders that might affect static and dynamic balance control was performed according to previous studies and accessibility data from the NPFSIT database. Respondents were surveyed on sociodemographic variables, including age, gender (male, female), education level (elementary school or lower, junior or senior school, and college or higher), monthly income level (≦20,000 NTD, 20,001–40,000 NTD, and ≧40,001 NTD), marital status (never married, married, and divorced/separation/widowed), relationship status (living with someone, not living with someone), and weekly regular exercise level (≧150 min/week, < 150 min/week). Anthropometric variables included height, body weight, BMI, WC, HC, and WHR. Height, body weight, WC and HC were measured and recorded by trained study assistants according to NPFSIT anthropometric standard procedures (31). BMI was calculated as the participant’s body weight in kilograms divided by the square of height in meters. WHR was calculated as the participant’s WC in centimeters divided by the HC in centimeters. Obesity was classified into two categories: general and abdominal obesity. According to the Taiwan’s Health Promotion Administration of Ministry of Health and Welfare guidelines (32), the normative values for general obesity was defined as underweight (BMI < 18.5 kg/m^2^), normal (18.5 kg/m^2^ ≦ BMI < 24.0 kg/m^2^), overweight (24.0 kg/m^2^ ≦ BMI < 27.0 kg/m^2^) and obese (BMI ≧ 27.0 kg/m^2^). The normative values for abdominal obesity were defined either way: (1) WC ≥ 90 cm for males or WC ≥ 80 cm for females; (2) WHR ≥ 0.90 for males or WHR ≥ 0.85 for females according to the Taiwan’s Health Promotion Administration of Ministry of Health and Welfare guidelines (32).

### Statistical analysis

The software used to perform the statistical analysis for this study was SAS, version 9.4 (SAS Institute, Cary, NC, United States). For descriptive statistics, characteristics of the study participants were compared among lower-limb muscle strength quartiles with the chi-square test for categorical variables and one-way analysis of variance (ANOVA) for continuous variables. When a significant *F* value was found (*p* < 0.05), Tukey’s *post hoc* test was performed to determine the differences between the pairs of means. To examine the dose–response relationship of lower-limb muscle strength performance with static and dynamic balance control measurements, we established four different categories (quartiles) for the 30-s chair stand measurement. The lowest quartile (the reference group) comprised participants who performed the worst in the 30-s chair stand measurement. Multiple linear regression models were used to determine the associations of the continuous or categorical (quartiles) variables in lower-limb muscle strength performance with static and dynamic balance control measurements, and the results are presented as *β* coefficients. All values are expressed as the mean ± standard deviation (SD) or the percentage. For all the analyses, a *p* value of <0.05 was considered statistically significant.

## Results

Table 2 shows the association of continuous or categorical (quartiles) variables in lower extremity muscle strength performance with static and dynamic balance control measures. For both genders, continuous variables in lower extremity muscle strength performance were significantly associated with static and dynamic balance control measures. Moreover, whether the participant was male or female, after adjusting for BMI, education, monthly income, and marital and relationship status, there was still significant, positive regression coefficient values in static balance measures (Men: *β* = 0.553, *p* ≤ 0.0001; Women: *β* = 0.503, *p* ≤ 0.0001) and negative regression coefficient values in dynamic measures (Men: *β* = −0.206, *p* ≤ 0.0001; Women: *β* = −0.222, *p* ≤ 0.0001).

**Table 2:** Multiple regressions for the associations of the continuous or categorical (quartiles) variables in lower-limb muscle strength performance with static and dynamic balance control measurements after adjustment for potential confounders.

Variables	Lower-limb muscle strength performance	Model 1^a^				Model 2^b^			
		*β*	SE	*p*	Partial eta squared	*β*	SE	*p*	Partial eta squared
**Men**									
*Static balance*	Continuous variable	0.667	0.023	< 0.0001*	0.111	0.553	0.026	< 0.0001*	0.079
One-leg stance with eye open test (s)	Categorical variable								
	Q4	9.295	0.356	< 0.0001*	0.085	7.700	0.394	< 0.0001*	0.059
	Q3	5.325	0.377	< 0.0001*	0.026	4.590	0.405	< 0.0001*	0.021
	Q2	2.723	0.360	< 0.0001*	0.008	2.539	0.385	< 0.0001*	0.007
	Q1	Ref.	—	—		Ref.	—	—	
Test for trend		*p* < 0.0001*				*p* < 0.0001*			
*Dynamic balance*	Continuous variable	−0.225	0.004	< 0.0001*	0.327	−0.206	0.005	< 0.0001*	0.295
8-foot up-and-go test (s)	Categorical variable								
	Q4	−3.370	0.067	< 0.0001*	0.255	−3.091	0.073	< 0.0001*	0.224
	Q3	−2.601	0.071	< 0.0001*	0.154	−2.434	0.075	< 0.0001*	0.145
	Q2	−1.755	0.068	< 0.0001*	0.083	−1.661	0.072	< 0.0001*	0.080
	Q1	Ref.	—	—		Ref.	—	—	
Test for trend		*p* < 0.0001*				*p* < 0.0001*			
**Women**									
*Static balance*	Continuous variable	0.645	0.018	< 0.0001*	0.094	0.503	0.020	< 0.0001*	0.063
One-leg stance with eye open test (s)	Categorical variable								
	Q4	8.745	0.273	< 0.0001*	0.071	6.920	0.305	< 0.0001*	0.047
	Q3	5.291	0.259	< 0.0001*	0.030	4.179	0.284	< 0.0001*	0.021
	Q2	2.388	0.268	< 0.0001*	0.006	2.022	0.291	< 0.0001*	0.005
	Q1	Ref.	—	—		Ref.	—	—	
Test for trend		*p* < 0.0001*				*p* < 0.0001*			
*Dynamic balance*	Continuous variable	−0.243	0.003	< 0.0001*	0.324	−0.222	0.004	< 0.0001*	0.287
8-foot up-and-go test (s)	Categorical variable								
	Q4	−3.517	0.052	< 0.0001*	0.252	−3.196	0.060	< 0.0001*	0.218
	Q3	−2.729	0.050	< 0.0001*	0.183	−2.548	0.055	< 0.0001*	0.170
	Q2	−1.773	0.052	< 0.0001*	0.081	−1.660	0.057	< 0.0001*	0.076
	Q1	Ref.	—	—		Ref.	—	—	
Test for trend		*p* < 0.0001*				*p* < 0.0001*			

*β*, regression coefficient; BMI, body mass index; Quartile 1, Q1; Quartile 2, Q2; Quartile 3, Q3; Quartile 4, Q4; SE, standard error. *p < 0.05.

a Adjusted for age.

b Adjusted for age, BMI, educational levels, monthly income levels, and marital and relationship status.

For both genders, categorical (quartiles) variables in lower extremity muscle strength performance were significantly associated with static and dynamic balance control measures. Increased lower-limb muscle strength levels were significantly associated with static balance in men (Q2: *β* = 2.539, *p* ≤ 0.0001; Q3: *β* = 4.590, *p* ≤ 0.0001; Q4: *β* = 7.700, *p* ≤ 0.0001) and women (Q2: *β* = 2.022, *p* ≤ 0.0001; Q3: *β* = 4.179, *p* ≤ 0.0001; Q4: *β* = 6.920, *p* ≤ 0.0001) after adjusting for potential confounders. In addition, we observed that increased lower-limb muscle strength levels were significantly associated with dynamic balance in men (Q2: *β* = −1.661, *p* ≤ 0.0001; Q3: *β* = −2.434, *p* ≤ 0.0001; Q4: *β* = −3.091, *p* ≤ 0.0001) and women (Q2: *β* = −1.660, *p* ≤ 0.0001; Q3: *β* = −2.548, *p* ≤ 0.0001; Q4: *β* = −3.196, *p* ≤ 0.0001) after adjusting for potential confounders.

## Discussion

In this study, we analyzed the relationship between lower-limb muscle strength performance and static and dynamic balance control using data from 20,846 Taiwanese older adult adults. The main findings of this study were as follows: First, lower-limb muscle strength was positively associated with static and dynamic balance performance, and we also observed dose–response relationships in the aforementioned variables; and second, higher levels of lower-limb muscle strength were associated with higher regular exercise and a lower risk of abdominal obesity. These findings suggest that older adult adults with good static and dynamic balance and quick walking speeds are more independent and have a reduced risk of sarcopenia and falling.

It has been reported that adults who perform ≥150 min/week of moderate-intensity physical activity (e.g., cleaning, gardening, walking and yoga), which is associated with lower rates of cardiovascular diseases and premature mortality, are considered regular exercisers (33–35). In addition, Chen et al. found that higher levels of relative grip strength were associated with a lower risk of abdominal obesity and cardiorespiratory fitness (36). In this study, we observed that approximately 75% of older adults were classified as nonregular exercisers in Taiwan, and higher levels of nonregular exercise were associated with lower 30-s chair stand performance and higher body mass index and waist-to-hip ratio. Our findings suggested that older adult adults who exercise regularly may have better lower extremity muscle strength performance and are less at risk for abdominal obesity compared to those who do not exercise regularly. Consistent with previous findings, the results of this study suggest that individuals with weaker leg strength have a higher rate of risk for disease (e.g., metabolic syndrome) (37, 38).

A previous study indicated that older fallers are characterized by lower muscular strength and aerobic endurance performance (chair stand test and 6-min walk test, respectively) than nonfalling older adult men, whereas agility and dynamic balance (8-foot up-and-go test) tended to differ (*p* = 0.075) between the two groups (8). These disparate results could be the result of the sizes of and differences in sample sizes (fallers, *n* = 16 vs. nonfallers, *n* = 50). In the present study, we found that lower-limb muscle strength was positively associated with one-leg stance with the eye open test and 8-foot up-and-go test performance in both genders. A similar study indicated that a one-leg stance with the eye open test was associated with hip fracture risk; for example, the risk of hip fracture was 5% lower with a 1-s longer one-leg stance with the eye open test (39). We suggest that older adult adults with good lower-extremity muscle strength (30-s chair stand test, ≥ 12 repetitions) could increase static and dynamic balance, as well as prevent falling, thereby reducing the risk of fracture and improving quality of life.

Notably, this study is the first to examine the dose–response relationships between lower-limb muscle strength with the one-leg stance with the eye open test and the 8-foot up-and-go test after adjusting for confounding factors (e.g., age, BMI, educational levels, monthly income levels) among Taiwanese older adults. We observed that higher levels of lower-limb muscle strength were associated with higher agility and better static and dynamic balance performance in both genders. In addition, this study took as its starting point the global goal of healthy aging, which makes the study of the health of older adults in Taiwan a critical issue given that Taiwan will become a super-aged society by 2025 and the proportion of older adults in the population will increase more rapidly than in other countries.

The present study has some limitations. First, our questionnaire did not include fall history, physical activity, cardiovascular disease, hypertension or sarcopenia, which could interfere with muscle strength, balance and mobility performance. Second, this study was a cross-sectional study, and no cause and effect relationships can be guaranteed. Future studies should be conducted with a longitudinal study design to understand the cause and effect relationship between thigh muscle strength and static and dynamic balance performance in Taiwanese older adult individuals.

## Conclusion

This study demonstrated that lower-limb muscle strength was the most important factor, as it was an improved method for static and dynamic balance control in both genders. As Taiwan has one of the highest aging rates in the world, there is a high demand for comprehensive aging-related policies. Therefore, by providing useful information on personal balance and fall risk to guide the development of appropriate prevention and rehabilitation programs, this study allows older adults to self-assess their balance by conducting tests and taking appropriate actions to reduce fall risk, such as engaging in appropriate exercise and balance training.

## Data availability statement

The datasets presented in this study can be found in online repositories. The names of the repository/repositories and accession number(s) can be found in the article/supplementary material.

## Ethics statement

The studies involving humans were approved by Institutional Review Board of Fu Jen Catholic University (FJU-IRB C110113). The studies were conducted in accordance with the local legislation and institutional requirements. The participants provided their written informed consent to participate in this study.

## Author contributions

P-CY and T-SL participated in the design, conducted the statistical analyses, and interpreted the data, drafted and revised the manuscript. D-KS supervised the study, assisted in data interpretation, and critically reviewed the manuscript. C-CH helped to manage and analyze the data. All authors contributed to the article and approved the submitted version.
